# Wearing graduated compression stockings augments cutaneous vasodilation but not sweating during exercise in the heat

**DOI:** 10.14814/phy2.13252

**Published:** 2017-05-08

**Authors:** Naoto Fujii, Toshiya Nikawa, Bun Tsuji, Glen P. Kenny, Narihiko Kondo, Takeshi Nishiyasu

**Affiliations:** ^1^ Institute of Health and Sport SciencesUniversity of TsukubaTsukuba CityJapan; ^2^ Human and Environmental Physiology Research UnitUniversity of OttawaOttawaCanada; ^3^ Faculty of Human Culture and SciencePrefectural University of HiroshimaHiroshimaJapan; ^4^ Faculty of Human DevelopmentKobe UniversityKobeJapan

**Keywords:** Baroreceptors, Central blood volume, compression garments, heat stroke, thermoregulation

## Abstract

The activation of cutaneous vasodilation and sweating are essential to the regulation of core temperature during exercise in the heat. We assessed the effect of graduated compression induced by wearing stockings on cutaneous vasodilation and sweating during exercise in the heat (30°C). On two separate occasions, nine young males exercised for 45 min or until core temperature reached ~1.5°C above baseline resting while wearing either (1) stockings causing graduated compression (graduate compression stockings, GCS), or (2) loose‐fitting stockings without compression (Control). Forearm vascular conductance was evaluated by forearm blood flow (venous occlusion plethysmography) divided by mean arterial pressure to estimate cutaneous vasodilation. Sweat rate was estimated using the ventilated capsule technique. Core and skin temperatures were measured continuously. Exercise duration was similar between conditions (Control: 42.2 ± 3.6 min vs. GCS: 42.2 ± 3.6 min, *P *= 1.00). Relative to Control, GCS increased forearm vascular conductance during the late stages (≥30 min) of exercise (e.g., at 40 min, 15.6 ± 5.6 vs. 18.0 ± 6.0 units, *P *= 0.01). This was paralleled by a greater sensitivity (23.1 ± 9.1 vs. 32.1 ± 15.0 units°C^−1^, *P *= 0.043) and peak level (14.1 ± 5.1 vs. 16.3 ± 5.7 units, *P *= 0.048) of cutaneous vasodilation as evaluated from the relationship between forearm vascular conductance with core temperature. However, the core temperature threshold at which an increase in forearm vascular conductance occurred did not differ between conditions (Control: 36.9 ± 0.2 vs. GCS: 37.0 ± 0.3°C, *P *= 0.13). In contrast, no effect of GCS on sweating was measured (all *P* > 0.05). We show that the use of GCS during exercise in the heat enhances cutaneous vasodilation and not sweating.

## Introduction

The application of positive pressure to the lower limbs while in the upright posture increases venous return thereby enhancing cardiac output (Nishiyasu et al. 1998, 2007). A comparable response can be achieved with the use of commercially available graduated compression stockings (GCS) (Liu et al. 2008). These stockings are used for the management of vein disorders such as venous thrombosis and varicose veins, and have increasingly been employed by athletes and general population engaged in exercise training, to improve exercise performance and/or enhance the restoration of physiological function following exercise (e.g., reducing muscle soreness) (Ali et al. 2007; Goh et al. 2011; Bovenschen et al. 2013; Priego et al. 2015). It remains unclear, however, how the use of GCS affects heat loss responses during exercise especially in the heat.

At the onset of exercise, there is a rapid increase in rate of metabolic heat production with stable levels achieved within the first few minutes of exercise. However, it is not immediately matched by a similar increase in the rate of heat loss thus giving rise to a pronounced increase in body heat storage, and therefore body core temperature during the early stages of exercise. The swift and sustained activation of heat loss responses of cutaneous vasodilation and sweating during exercise is therefore necessary to prevent potentially dangerous increases in body core temperature. Previous studies have shown that during heat stress such as passively heated resting or exercise in the heat, an increase in venous return or central blood volume can enhance cutaneous perfusion, a response which has been ascribed to an increase in baroreceptor loading status (Nielsen et al. 1984; Nose et al. 1990; Nagashima et al. 1998; Crandall et al. 1999; Gonzalez‐Alonso et al. 1999; Schlader et al. 2015; Paull et al. 2016). In contrast, although some studies reported that baroreceptors may also modulate the heat loss response of sweating during (Mack et al. 1988, 2001) and following (Gagnon et al. 2008) dynamic exercise, the results are less conclusive with some recent studies showing no effect during heat stress at rest (McGinn et al. 2014; Schlader et al. 2015; Paull et al. 2016). Indeed, we recently demonstrated that GCS augments cutaneous vasodilation, but not whole‐body sweating (i.e., as evidenced by similar changes in body weight – an indirect index of total sweat loss – between GCS and no compression stockings) during passive heating at rest (Fujii et al. 2017). However, in order to delineate the influence of GCS on sweating, the direct measurement of sweat rate is necessary. Also, given that heat loss responses differ between rest and exercise (Kondo et al. 2010; Simmons et al. 2011), the above results obtained at rest (Fujii et al. 2017) may not necessarily reflect responses during exercise in the heat.

Accordingly, in this study, we evaluated the hypothesis that stocking‐mediated graduated compression augments cutaneous vasodilation but not sweating during exercise in the heat.

## Materials and Methods

### Ethical approval

This study was approved by the Human Subjects Committee of the University of Tsukuba in agreement with the Declaration of Helsinki. Written informed consent was obtained from all subjects before their participation.

### Subjects

Nine young males participated in this study. Individuals with chronic diseases, taking prescription medication, or were smokers were excluded from the study. The subjects' age, height, body mass, and peak oxygen uptake, presented in mean ± standard deviation, were 24.7 ± 2.0 years, 1.72 ± 0.03 m, 66.3 ± 3.3 kg, and 52.0 ± 6.0 mL kg^−1^ min^−1^, respectively. All subjects were instructed to abstain from taking over‐the‐counter medications for at least 48 h prior to any experimental session, as well as alcohol and caffeine consumption for at least 12 h. Subjects also refrained from performing intense exercise the day prior to the study.

### Preliminary session

All subjects completed an incremental cycling bout until exhaustion to determine their peak oxygen uptake. A customized semirecumbent cycle ergometer (Model 818E, Monark, Stockholm, Sweden) was employed for the cycling. Initial work load was set at 60 W, which was increased every 1 min by 15 W with a constant pedaling rate of 60 rpm. The test continued until the subjects could no longer maintain a pedaling rate of >50 rpm or until volitional fatigue. Throughout the test, the subjects breathed from a mask, which covered the mouth and nose. A mass‐flow sensor (hot‐wire type) and a gas‐sampling tube (the sampling volume rate was below 0.2 L min^−1^) were connected to the mask, and the expired volume and gases were analyzed continuously using an electric gas flow meter (Model RM300i, Minato Medical Science, Japan). Peak oxygen uptake was determined as the highest value measured over a 1 min period. The test was performed in an environmental chamber (Fujiika, Chiba, Japan) regulated at 25°C and 50% relative humidity.

### Experimental session

Several days after completing the incremental maximal exercise test, the subjects performed an exercise‐heat stress test. This test consisted of two trials performed on separate days in a counter‐balanced manner (each separated by at least 4 days) wherein the participants exercised in the heat while wearing either: (1) graduate compression stockings (GCS) or (2) loose‐fitting stockings without compression (Control). The commercially available GCS was provided by ALCARE Limited (REGUARD CG Tights EX33, ALCARE, Tokyo, Japan). The stockings were water permeable, pantyhose type, covering the lower limbs including the feet with the exception of the toes and heels which remained exposed. Each participant was given their own custom‐fitted stockings as determined by the subject's body mass and circumference of both the waist and ankle. The same stockings were cut and loosened and used for the Control condition. Our recent work (Fujii et al. 2017) demonstrated that the GCS induced graduated pressures of 26.4 ± 5.3, 17.5 ± 4.4, and 6.1 ± 2.0 mmHg at the ankle (upper part of lateral malleolus), calf (head of fibula), and thigh (mid‐point of vastus lateralis), respectively, whereas the loose‐fitting stockings yielded negligible pressures at each skin site (*n *= 9). In this work, we applied the pressure sensors (AMI3037‐10, AMI Techno, Tokyo, Japan) underneath the stockings at the same locations on the right limb as described above. These measurements were performed during rest in a room maintained at 30°C and 50% relative humidity (Fuji Medical Science Co., Ltd).

To ensure that participants were euhydrated prior to the start of the experimental trial, they were instructed to drink 500 mL of water the night before. Further, on the day of the experiment, the subjects consumed only a light breakfast and 300 mL of water at least 2 h before each trial. Upon arrival at the laboratory at 9:00 am, the subjects voided their bladder, thereafter body mass with shorts only was measured using a weight scale platform. They then donned either the GCS or loose‐fitted stockings, and entered the environmental chamber regulated to 30°C and relative humidity of 50% (Fuji Medical Science Co., Ltd, Chiba, Japan). They rested in a semirecumbent position in the chamber for 60 min during which time they were instrumented. The participants then performed semirecumbent cycling at 60% peak oxygen uptake at a pedaling rate of 60 rpm for 45 min or until body core temperature reached ~1.5°C above baseline resting values. Following the exercise, postexercise body mass was recorded.

## Measurements

Esophageal and skin temperatures were obtained every 1 sec using self‐made copper constantan thermocouples, the information of which was stored in a computer through a data logger system (WE7000, Yokogawa, Tokyo, Japan). Esophageal temperature was measured using a temperature probe inserted via the nostril and positioned in the esophagus at a depth equivalent to one quarter of the subject's height. Skin temperature was recorded at seven sites (forehead, forearm, hand, feet, upper calf, front thigh, and chest), and mean skin temperature was calculated using the method of Hardy and Dubois (1938).

Forearm blood flow was evaluated every 30 sec using venous occlusion plethysmography with the aid of a mercury‐silastic strain gauge (Whitney 1953). Increases in forearm blood flow in response to heat stress reflects primarily increases in skin blood flow, since forearm muscle blood flow remains relatively unchanged during exercise (Johnson and Rowell 1975). The forearm was supported and elevated approximately 0.1 m above heart level by slings to maintain venous return. Pressure in the venous occlusion cuff was maintained at ~45 mmHg, while the circulation to the hand was restricted using a wrist cuff inflated to 220–230 mmHg. Forearm blood flow was recorded twice each minute for a minimum of 10–20 sec after the upper arm cuff was inflated. The wrist cuff was released for ~1 min every 10 min so as not to cause undue physical discomfort to the subject. During this period, forearm blood flow was not measured. To account for the influence of changes in perfusion pressure on blood flow, forearm vascular conductance was evaluated as the forearm blood flow divided by mean arterial pressure (diastolic arterial pressure plus one‐third of the pulse pressure). Arterial blood pressures were measured every 1 min via an automated sphygmomanometer (STBP‐780; Nippon Colin, Tokyo, Japan). In order to calculate forearm vascular conductance, a single mean arterial pressure measurement was used for each two corresponding forearm blood flow recordings obtained in each 1‐min period. Heart rate was recorded every 5 sec via a heart rate monitor (HR monitor Vantage NV, POLAR, Kempele, Finland).

Chest sweat rate was measured using the ventilated‐capsule method. Nitrogen gas was supplied at a rate of 1.2 L min^−1^ to a capsule attached to the chest skin (3.5 cm^2^). The humidity in the nitrogen gas obtained from the capsule was then measured by a capacitance hygrometer (HMP 45ASPF, Vaisala, Helsinki, Finland), the information of which was used to calculate sweat rate. Values were averaged over 30‐sec periods.

From the gas analyzers (Minato Medical Science), minute ventilation, tidal volume, respiratory frequency, end‐tidal CO_2_ pressure, O_2_ uptake, CO_2_ output, and respiratory exchange ratio were recorded over 30‐sec periods. Arterial CO_2_ pressure was estimated using tidal volume and end‐tidal CO_2_ pressure according to the equation of Jones et al. (1979).

During the exercise, 6–20 point ratings of perceived exertion (Borg 1975) were recorded every 5 min.

## Data analyses

**Figure 1:**
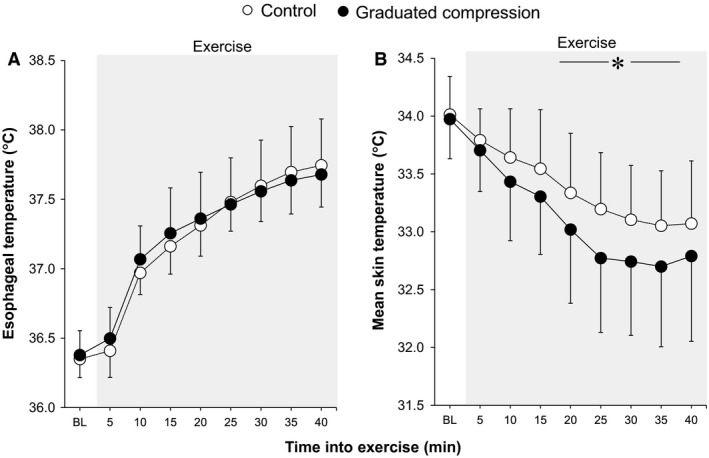
Time‐course changes in esophageal (A) and mean skin (B) temperatures during baseline resting and exercise. *between conditions (*P* ≤ 0.05). Data are mean ± standard deviation (*n *= 9). BL, baseline.

**Figure 2:**
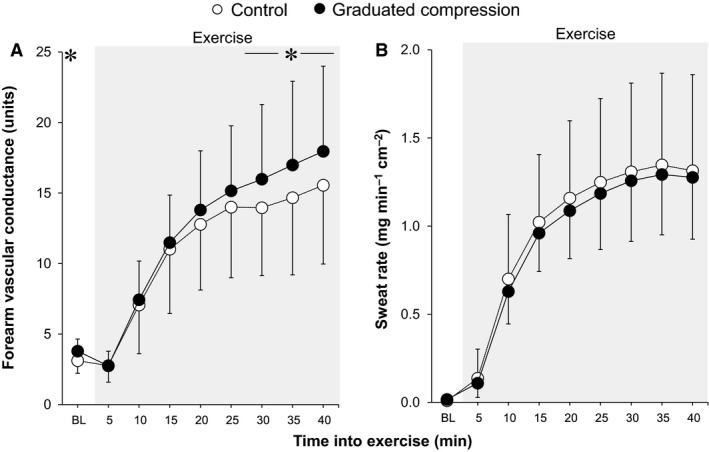
Time‐course changes in forearm vascular conductance (A) and sweat rate (B) during baseline resting and exercise. *between conditions (*P* ≤ 0.05). Data are mean ± standard deviation (*n* = 9). BL, baseline.

**Figure 3:**
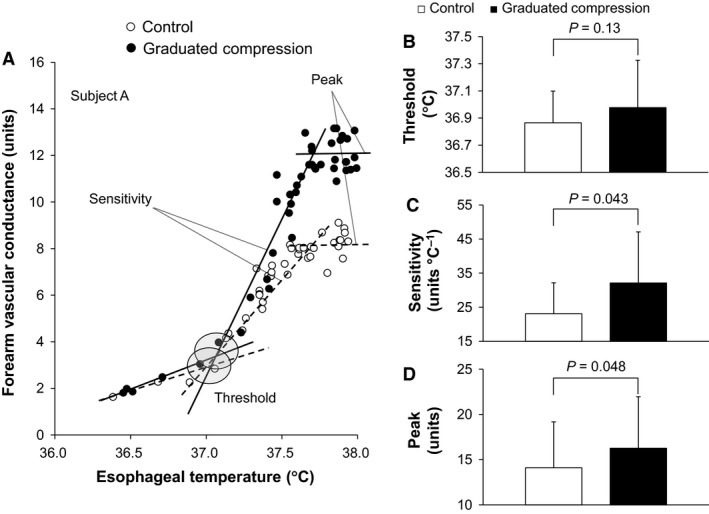
A representative individual data for forearm vascular conductance plotted against esophageal temperature (A). Averaged esophageal temperature threshold (B), sensitivity (C), and peak (D) for cutaneous vasodilation are also presented. Data are mean ± standard deviation (*n *= 9) for Figure B, C, and D. Units for forearm vascular conductance are ml 100 mL tissue^−1^ min^−1^ mm Hg *100.

**Figure 4:**
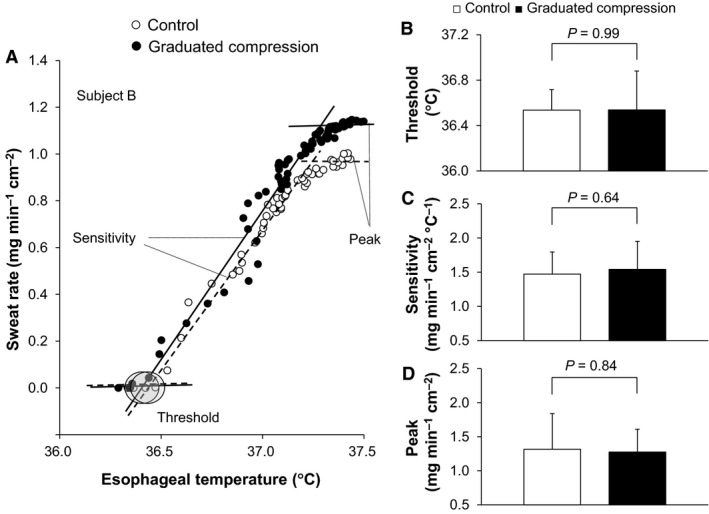
A representative individual data for sweat rate plotted against esophageal temperature (A). Averaged esophageal threshold (B), sensitivity (C), and peak (d) for sweat rate are also presented. Data are mean ± standard deviation (*n *= 9) for Figure B, C, and D.

All data used for time‐dependent data analysis (including Fig. 1 and 2 and Tables 1 and 2) were averaged over each 5‐min interval. As described in our previous work (Fujii et al. 2012b), the baseline horizontal line and the regression line were derived from the relationship between body core temperature and forearm vascular conductance using data averaged over 30 sec. The slope of the regression line was employed to determine the thermal sensitivity of the cutaneous vasodilatory response. The threshold for cutaneous vasodilation was defined as the body core temperature at which the onset of cutaneous vasodilation occurred as determined by the point at which the regression line crossed the baseline horizontal line. Peak value of cutaneous vasodilation was assessed by averaging the values over the last 5 min of exercise. A similar evaluation was also performed for sweating. Figures 3A and 4A illustrate these measurements.

**Table 1:** Time‐course changes in heart rate and mean arterial pressure

		Time into exercise (min)							
	BL	5	10	15	20	25	30	35	40
Heart rate, beats min^−1^									
Control	61 ± 8	125 ± 9	139 ± 11	142 ± 11	144 ± 11	147 ± 11	148 ± 11	151 ± 12	153 ± 13
Graduated compression	58 ± 7	126 ± 9	139 ± 9	141 ± 9	143 ± 9	145 ± 10	148 ± 11	150 ± 10	152 ± 10
Mean arterial pressure, mmHg									
Control	78 ± 5	94 ± 8	99 ± 6	96 ± 5	96 ± 5	95 ± 5	95 ± 5	95 ± 6	95 ± 6
Graduated compression	77 ± 5	96 ± 7	100 ± 7	97 ± 8	95 ± 7	94 ± 6	94 ± 5	92 ± 6	92 ± 6

Data are mean ± standard deviation (*n *= 9).

BL, baseline at rest. There was no between‐condition difference throughout for both heart rate and mean arterial pressure (all *P* > 0.05).

**Table 2:** Time‐course changes in respiratory variables

		Time into exercise (min)							
	BL	5	10	15	20	25	30	35	40
Minute ventilation, L min^−1^									
Control	10.0 ± 1.6	45.4 ± 5.3	54.4 ± 5.5	56.5 ± 5.1	59.0 ± 5.0	61.0 ± 5.6	62.6 ± 6.6	64.5 ± 6.7	65.3 ± 4.6
Graduated compression	9.7 ± 0.8	46.2 ± 2.7	54.3 ± 2.9	55.4 ± 3.7	56.7 ± 4.9	58.5 ± 4.5	59.2 ± 4.3	61.1 ± 4.6	61.6 ± 4.9
Tidal volume, L									
Control	0.6 ± 0.1	1.5 ± 0.2	1.6 ± 0.2	1.6 ± 0.2	1.6 ± 0.3	1.6 ± 0.3	1.6 ± 0.3	1.6 ± 0.3	1.5 ± 0.3
Graduated compression	0.6 ± 0.1	1.6 ± 0.2	1.7 ± 0.2	1.6 ± 0.2	1.6 ± 0.2	1.6 ± 0.3	1.6 ± 0.3	1.6 ± 0.4	1.5 ± 0.4
Respiratory frequency, breaths min^−1^									
Control	17 ± 3	31 ± 5	34 ± 6	36 ± 6	38 ± 9	40 ± 10	42 ± 10	43 ± 10	46 ± 10
Graduated compression	16 ± 2	30 ± 5	33 ± 6	35 ± 6	37 ± 8	38 ± 8	39 ± 8	41 ± 10	42 ± 10
Estimated arterial CO_2_ pressure, mmHg									
Control	40.1 ± 2.2	43.5 ± 2.5	42.4 ± 2.8	41.9 ± 2.7	41.1 ± 2.6	40.3 ± 2.3	40.0 ± 2.5	39.3 ± 2.5	38.9 ± 1.6
Graduated compression	42.1 ± 1.4^a^	44.3 ± 2.1	43.1 ± 2.1	42.4 ± 1.8	41.9 ± 1.6	41.4 ± 1.4	41.0 ± 1.3	40.6 ± 1.5^a^	40.4 ± 1.4^a^
O_2_ uptake, L min^−1^									
Control	0.27 ± 0.03	1.78 ± 0.24	2.02 ± 0.20	2.07 ± 0.19	2.11 ± 0.20	2.13 ± 0.20	2.16 ± 0.21	2.17 ± 0.20	2.18 ± 0.19
Graduated compression	0.27 ± 0.02	1.84 ± 0.17	2.06 ± 0.18	2.06 ± 0.18	2.06 ± 0.18	2.11 ± 0.18	2.11 ± 0.17	2.15 ± 0.16	2.14 ± 0.15
CO_2_ output, L min^−1^									
Control	0.24 ± 0.03	1.65 ± 0.21	1.93 ± 0.17	1.96 ± 0.18	2.00 ± 0.18	2.00 ± 0.17	2.02 ± 0.20	2.04 ± 0.20	2.01 ± 0.13
Graduated compression	0.24 ± 0.02	1.73 ± 0.14	1.98 ± 0.18	1.95 ± 0.18	1.95 ± 0.18	1.99 ± 0.18	1.98 ± 0.17	2.00 ± 0.16	1.98 ± 0.15
Respiratory exchange ratio									
Control	0.88 ± 0.06	0.93 ± 0.05	0.96 ± 0.03	0.95 ± 0.03	0.95 ± 0.03	0.94 ± 0.03	0.94 ± 0.03	0.94 ± 0.04	0.92 ± 0.04
Graduated compression	0.88 ± 0.02	0.94 ± 0.05	0.96 ± 0.03	0.95 ± 0.02	0.94 ± 0.02	0.94 ± 0.02	0.94 ± 0.02	0.93 ± 0.03	0.93 ± 0.03

Data are mean ± standard deviation (*n *= 9). BL, baseline at rest.

a vs. Control (*P* ≤ 0.05).

## Statistical analyses

Data are presented as mean ± standard deviation. Time‐dependent data were analyzed using a two‐way repeated‐measures analysis of variance with two factors of condition (Control and GCS) and time (baseline and every 5 min of the 40‐min exercise bout). For the time‐dependent analysis, data for nine subjects were included for all time points with the exception of the 40 min of exercise whereby only eight out of the nine subjects completed the final 5 min. When a main effect or an interaction was detected, post hoc multiple comparisons were performed using two‐tailed paired *t*‐tests for the between‐condition difference with *P* value adjusted in order to keep alpha level of *P* ≤ 0.05. Two‐tailed paired *t*‐tests were also used to compare variables between conditions where applicable. Value of *P* ≤ 0.05 was considered statistically significant. Statistical analyses were performed using the software package SPSS 24 (IBM, Armonk, NY).

## Results

Total exercise time did not differ between the Control and GCS conditions (42.2 ± 3.6 vs. 42.2 ± 3.6 min, *P *= 1.00). A similar increase in esophageal temperature was measured during exercise in both conditions (all *P* > 0.15, Fig. 1A). Forearm vascular conductance at baseline and between 30 and 40 min of exercise was greater in the GCS in comparison to the Control conditions (all *P* ≤ 0.05, Fig. 2A). This greater exercise‐induced forearm vascular conductance was paralleled by an elevated sensitivity and peak of cutaneous vasodilation as evaluated from the relationship between forearm vascular conductance and esophageal temperature (both *P* ≤ 0.05, Fig. 3C, D). In contrast to cutaneous vasodilatory responses, there were no between‐condition differences in sweat rate during baseline resting or throughout exercise (all *P* > 0.76, Fig. 2B). Similarly, no differences were observed in the threshold and sensitivity of the sweating response as well as peak sweat rate in relation to the change in esophageal temperature (all *P* > 0.64, Fig. 4). A lower mean skin temperature was measured between 20 and 35 min of exercise in the GCS as compared to the Control condition (Fig. 1B). This was primarily associated with a ~2.5°C lower calf temperature (all *P* ≤ 0.05) as no differences were observed between conditions for the other skin sites (all *P* > 0.05).

Heart rate and mean arterial pressure did not differ between conditions throughout the experiment (all *P* > 0.20, Table 1). Similarly, no differences in respiratory responses were observed between conditions (all *P* > 0.05) with the exception that a higher estimated arterial CO_2_ pressure at rest and at 35 and 40 min of exercise (all *P* ≤ 0.05) and a lower tendency of minute ventilation during the latter period of exercise (*P *= 0.12, 0.12, and 0.06 for 30, 35, and 40 min into exercise, respectively) in the GCS relative to the Control condition (Table 2). A similar reduction in body weight following exercise was measured between conditions (Control: 1.1 ± 0.5 vs. GCS: 1.0 ± 0.2 kg, *P *= 0.69). Rating of perceived exertion during exercise did not differ between conditions (all *P* > 0.24).

## Discussion

We demonstrated for the first time that graduated compression associated with the use of commercially available stockings induced increases in cutaneous vasodilation during exercise in the heat. This response was associated with an increased sensitivity and peak level of the cutaneous vasodilation response as evaluated from the relation of body core temperature with cutaneous perfusion. In contrast to cutaneous vasodilation, no effect on the sweating response was observed with the use of graduated compression stockings.

### Cutaneous vasodilation

We observed that GCS augmented cutaneous vasodilation during resting and exercise in the heat (Fig. 2A). The latter was the result of both an increase in the sensitivity of the response and an elevation in the peak response attained for a given change in body core temperature and not due to differences in the onset threshold for cutaneous vasodilation (Fig. 3). Similar results were also obtained in our recent study using passive heat stress at rest (Fujii et al. 2017). This augmentation of cutaneous vasodilation is likely a consequence of a compression stockings‐induced modulation of baroreceptor loading status brought about by the increase in venous return. In support of this possibility, increases in venous return and central blood volume due to postural manipulation (e.g., upright to supine) has been shown to augment cutaneous vasodilation during heat stress under resting or exercising conditions (Johnson et al. 1974; Gonzalez‐Alonso et al. 1999). A similar pattern of response during rest or exercise has been observed with the application of positive pressure to the lower limbs (McGinn et al. 2014; Paull et al. 2016), infusion of saline (Nose et al. 1990; Crandall et al. 1999; Schlader et al. 2015), head‐up water immersion (Nielsen et al. 1984), and continuous negative‐pressure breathing (Nagashima et al. 1998).

In addition to increased venous return, the higher arterial CO_2_ pressure associated with wearing GCS (Table 2) may in part explain the augmented cutaneous vasodilation. Indeed, changes in arterial CO_2_ can mediate changes in local vascular tone. For example, it has been shown that hypercapnia can induce cutaneous vasodilation during normothermic rest (Simmons et al. 2007) while hypocapnia can attenuate cutaneous vasodilation during heat stress at rest (Fujii et al. 2012a) and exercise in the heat (Fujii et al. 2014), though the effect of CO_2_ on cutaneous circulation is not always observed (Wingo et al. 2008). The precise mechanism(s) by which GCS induced a higher arterial CO_2_ pressure remains unclear. It is plausible, however, that the slight reduction in minute ventilation with GCS during the latter phase of exercise may have contributed to the higher arterial CO_2_ pressure by attenuating CO_2_ gas exchange. However, it should be noted that the higher arterial CO_2_ pressure was also detected prior to the start of exercise during the baseline resting period wherein minute ventilation was comparable between conditions (Table 2). This suggests that other factors are likely involved in the modulation of arterial CO_2_ pressure with the use of GCS. Future investigations are required to elucidate underlying mechanisms.

Our results showed that the use of GCS reduced mean skin temperature (Fig. 1B), albeit this was primarily due to the marked reductions in lower calf skin temperature (i.e., ~2.5°C). This lower calf skin temperature potentially reflects a reduced cutaneous perfusion. During the occlusion of the leg using an inflatable cuff, venous pressure increases to similar levels of cuff pressure (Christ et al. 1997). In our recent experiment, we measured calf pressure of 17.5 ± 4.4 mmHg under the GCS (Fujii et al. 2017). As such, calf venous pressure in the current experiment may have increased to a similar extent, reducing perfusion pressure thereby attenuating the increase in cutaneous blood flow. Noteworthy, thigh skin temperature was not similarly influenced by the compression stockings despite an increased venous pressure due to the GCS. Perhaps, the GCS‐induced thigh pressure of 6.1 ± 2.0 mmHg, measured in our recent experiment (Fujii et al. 2017), was not high enough to restrict cutaneous blood flow. Further study is warranted to elucidate the relationship between compression pressure and cutaneous perfusion under stockings.

### Sweating

In contrast to cutaneous vasodilation, no effect of GCS was observed on the sweating response at rest or during exercise in the heat (Figs 2B, 4B, C, D). In keeping with this, previous reports showed that increases in central blood volume associated with saline infusion (Schlader et al. 2015) and application of positive pressure to the lower limbs (McGinn et al. 2014; Schlader et al. 2015; Paull et al. 2016) have no effect on sweating during heat stress at rest. While our measurements of local sweat rate were limited to the chest only, it is likely that this response reflects whole‐body sweating as we found no between‐condition difference in the reduction in body weight following exercise.

### Heart rate and mean arterial pressure

Heart rate and mean arterial pressure were similar during rest and throughout the exercise period (Table 1). This is consistent with previous results (Iwama 1995; Houghton et al. 2009; Rimaud et al. 2010; Lucas et al. 2012; Morrison et al. 2014; Priego Quesada et al. 2015), though lower mean arterial pressure may occur during heat stress at rest (Morrison et al. 2014). Our observation that mean arterial pressure did not differ between conditions is noteworthy given that a greater cutaneous vasodilation can lead to a reduction in total peripheral resistance and therefore mean arterial pressure. It may be that the GCS increased cardiac output without influencing heart rate, thus counteracting the cutaneous vasodilatory effect. The lack of an effect of GCS on heart rate is itself interesting since an increase in venous return is known to lower heart rate. Further studies are required to elucidate the mechanisms underpinning these responses.

### Limitations

We tested males only in this study. Thus, our results cannot be simply applied to young females. Future studies are warranted to evaluate if there are sex‐related differences in the effect of GCS on cutaneous vasodilation and sweating during exercise in the heat. In this study, we assessed the influence of GCS on heat loss responses while the participant exercised in a semirecumbent position. Had the participants performed exercise in the upright position such as during running or upright cycling, changes in venous return would have differed thereby potentially affecting heat loss responses differently. Finally, it is important to note that we did not measure cardiovascular parameters such as cardiac output, total peripheral resistance, limb blood flow and other variables that could provide additional information pertaining to the effect of GCS on cardiovascular responses. However, it is not likely that GCS greatly affects active muscle blood flow, since oxygen uptake during exercise did not differ between conditions (Table 2). Also, we assessed heat loss responses at one skin site region only. Therefore, we are unable to determine if the GCS modulated heat loss responses at other skin sites, if at all.

### Perspectives and significance

Previous studies showed that the use of GCS is effective in preventing deep venous thrombosis (Coleridge Smith et al. 1991; Ibegbuna et al. 1997). Additionally, they have been shown to improve exercise performance during both distance running (Kemmler et al. 2009) and maximal cycling (Chatard et al. 2004), albeit these are not universal findings (Ali et al. 2007; Davies et al. 2009). Our results provide new insights into the possibility that GCS may also enhance heat dissipation. However, we show that this was not paralleled by differences in body core temperature. This latter response is consistent with previous findings that reported no effect of compression stockings on body core temperature during exercise performed in cool to hot ambient temperatures (i.e., 10 and 35°C) (Houghton et al. 2009; Goh et al. 2011; MacRae et al. 2012; Noonan and Stachenfeld 2012; Barwood et al. 2013). Wearing the compression stockings can insulate the skin thereby attenuating heat loss in the region covered by the stockings (Doan et al. 2003; Born et al. 2013; Engel et al. 2016). This may in part offset any increase in whole‐body heat dissipation induced by the GCS thereby resulting in no change in body core temperature. GCS that uses materials with low insulative properties may be required to maximize local heat loss. Further, it would be important to evaluate whether improved heat loss associated with wearing GCS enhances exercise performance in the heat. In addition, our results suggest that GCS induces a higher arterial CO_2_ pressure at rest and during late stages of exercise in the heat. Since arterial CO_2_ pressure is tightly coupled with cerebral blood flow during exposure to a heat stress at rest (Fujii et al. 2008; Brothers et al. 2009; Nelson et al. 2011; Bain et al. 2013) and during exercise in the heat (Nybo and Nielsen 2001; Rasmussen et al. 2006; Hayashi et al. 2011; Tsuji et al. 2015), the higher arterial CO_2_ pressure may lead to higher cerebral blood flow, which requires further scrutiny.

## Conclusions

Our results suggest that graduated compression achieved by wearing compression stockings augments cutaneous vasodilation but not sweating during exercise in the heat. We show that the augmented cutaneous vasodilation is due to increased sensitivity and peak level of cutaneous vasodilation and not a change in the onset threshold for cutaneous vasodilation.

## Conflict of Interest

None decalred.
